# Pelvic Abscess in the Female

**Published:** 1885-07

**Authors:** William Warren Potter

**Affiliations:** Buffalo, N. Y.; 306 Franklin Street


### BUFFALO

### Medical and Surgical Journal.

Vol. XXV. JULY, 1885. No. 12.

### Original Communications.

### Pelvic Abscess in the Female.

By William Warren Potter, M. D., Buffalo, N. Y.

In presenting to the association the subject for discussion this evening, which has been announced on the programme, attention is respect lly invited to the following history of a case of acute pelvic abscess, which is related by way of introduction:

Mrs. A., aged 38 years; twice married; living with her second husband; mother of three children by first husband—never pregnant by the second; fell ill on the 17th of March with severe pain in the right illiac region. I saw her first on the 19th, two days after the attack, when her condition was noted as follows: decubitus dorsal, lower extremities flexed, severe pain in lower abdomen, nausea, temperature 100 F, pulse 100, abdominal muscles tense, but no distension. Per vaginam: uterus hanging somewhat low in the pelvic cavity but mobile, os patulous but smooth, bladder tender, rectum empty. Bi-manual palpation attempted, but abandoned on account of pain inflicted; vesical tenesmus extreme. Treatment: Morphia and atropia hypodermatically administered; oleate of morphia to abdomen; hot vaginal douche. Catheterization at regular intervals. Next day, condition much the same, excepting temperature 101 F, pulse 110. Pain still severe, unless under full influence of anodynes. Right broad ligament very tender.

For the next few days, or until the 26th—one week after my first visit—the patient was kept in a state of semi-narcosis, with a daily increase in the quantities of the drugs; the temperature had reached 102$^2$ F, pulse 120; abdomen somewhat distended and very tender; uterus pushed forward under pubic arch—pelvic impaction; and at night she had a severe chill. Fluctuation detected in post-uterine space; consultation sought, and Dr. Thomas Lothrop saw the case with me on the 27th. He confirmed the diagnosis of pelvic abcess, but advised a day's delay in invoking surgical interference. Accordingly, next day, the 28th, nine days after my first visit, the patient was placed under ether by my son, Dr. F. H. Potter, and, with the kindly assistance of Drs. Lothrop and Tremaine, I drew off with the aspirator more than three pints of feted pus from an abscess occupying the position of Douglas's pouch. The needle, a medium-sized one, was thrust through the roof of the vagina, behind the uterus in the median line, and after the pus was drawn, the cavity was washed out with a mercuric chloride solution, 1 to 3,000. The opening was next enlarged, a drainage tube inserted, the vagina filled with marine lint, and the patient placed in bed. She had no more pain of any consequence, but she was very feeble, and emaciated to an extreme degree. Two days after the operation, at my evening visit, I thought she was dying; there was no pulse at the wrists, and she lost consciousness for a time; but under the hypodermatic use of alcohol, brandy, and ether, successively and rapidly administered, she rallied a little, and soon passed out of immediate danger. Her food and medicine had been administered per rectum for some time before the operation, as the stomach would hold nothing but iced champagne. Now, however, stomachal ingestion was partially resumed, though with great caution, for the slightest over-feeding provoked vomiting.

The washing out of the pus cavity was carefully carried out, first with the mercuric chloride, 1 to 5,000, then with carbolic water; but neither controlled the feted odor, nor seemed to diminish the quantity of pus, which, at the end of five days, was still of a dirty brownish tinge, and horribly offensive. Finally, in boxing the compass for a method by which iodoform could be carried into the cavity, I came across a plan which Dr. David Prince, of Illinois, adopted for injecting it into the bladder in chronic cystitis. Accordingly, an emulsion of iodoform with starch was prepared after his formula, which I injected into the abscess cavity, after first washing it out with carbolic water. This was done on the 2d of April, five days after the operation, and from that moment the case began to improve. All fetor at once disappeared, and the quantity of pus rapidly diminished. The cavity was washed out several times a day with the carbolic water, and the iodoform emulsion which was injected twice daily, was retained in the sac by means of a pine plug fitted into the mouth of the drainage tube. This plan was continued until the 12th of April, when the flow of pus ceased, the sac had filled by granulation and the drainage tube was withdrawn. The pulse and temperature had now reached the normal level, and convalescence was fully established. A few weeks more and she seemed fully restored to health.

I have omitted to mention that for some weeks prior to the attack which we have been considering, this lady had been visiting a so-called clairvoyant doctor, under whose advice she had taken about two pints of a mixture of cotton root and gin, but of what strength I was unable to learn.

This history is condensed from full daily records of the case, many interesting details of which have been omitted, for the sake of economizing time.

Among the many interesting features which the case presented, the following seemed to me especially noteworthy:

1. The rapidity with which so large an abscess formed in the non-puerperal state.

2. The corresponding rapidity with which it filled by granulation and finally closed.

3. The unusually large quantity of pus found upon opening the abscess, and drawn off at a single sitting.

4. The rapid improvement following the injection of iodoform into the abscess sac.

5. Did the cotton root play any considerable part in causing the abscess ?

I am unable to offer any satisfactory answer as to etiology in this case. I never saw the woman before this illness, and consequently know nothing of her previous history, excepting such facts as were obtained during the conduct of the case, and which have already been given in abstract.

But following, as did the abscess, so soon after taking the cotton root mixture, there is ground for the suspicion that the medicine may have at least served as an exciting cause.

Interest in the subject of peri-uterine abscess, both acute and chronic, has of late grown apace. This is due largely, no doubt, to the improved methods of diagnosis of diseases of the pelvic cavity, which have followed the introduction of the Sims speculum, bi-manual palpation, and the aspirator. The topographical anatomy of the pelvis is no longer a sealed book. We are no longer groping in the dark, so to speak, as was the case a few years ago; but since Henle, Savage, Foster, Hart, and Ranney have given us the benefit of their labors, the female pelvis may be said to be one of the best topographically mapped regions of the body. The improvements in the pathology and surgery of this region have been equally marked. The importance of early recognition of diseases of the pelvic connective tissue has been pointed out, and urgently insisted upon by Emmet, and his observations and teachings have made a lasting impression upon the profession; especially that portion of it more particularly concerned with the diseases of women. Here we have a fruitful and interesting field of observation, for the real pathogenesis of a multitude of pelvic disorders in woman lies, I am very sure, in an unrecognized, and, therefore, frequently uncured cellulitis.

When we consider the manifold conditions which are constantly at work, singly and in groups, having a tendency to light up inflammatory action the areolar tissue of the pelvis, this asseveration will not appear strange nor exaggerated. The area of exposure is enormously large. This connective tissue dips into all the pelvic interspaces; surrounds, overlaps, or envelops, partially or completely, all the pelvic organs; is in intimate relation with the tubes and ligaments; the bladder, uterus, vagina and rectum are mutually dependent upon it for its cushion-like steadying support. It is permeated with blood-vessels and nerves interwoven and doubled upon themselves, until, in attempting to trace them, one becomes lost in the mazy labyrinth of vessel and fibre—so intricate is the net-work of vein, artery and nerve. This is a wise arrangement, in that it permits great mobility of the organs which receive their blood and nerve supply through the cellular tissue, without placing the vessels on the stretch, or otherwise diminishing their calibre, whereby the proper nutrition of the viscera would become impaired. But once let inflammation invade the structures, and the weakness of the plan becomes manifest in the blood-stasis which it permits.

The nidus of pelvic cellular inflammation may be, oftentimes, a mere trifle—a slight irritation arising from that unknown quantity which we so succinctly formulate in the expression "taking cold." Irritation produces hypervascularity—there is hyperæmia. Hyperæmia produces arterial tension—there is loss of tone. Loss of tone causes an increase of blood pressure—there is congestion. This fulness of the vessels creates an exaltation of nervous force—there is nerve turmoil. These phenomena produce dilatation of the arterioles—there is inflammation. The veins refuse to return the increased quantity of blood—there is true blood-stasis. A stasis of blood is quite likely to set up peri-uterine inflammation, which may end in suppuration—in pelvic abscess.

Or, the disease may be of puerperal origin, following labor at term, or an abortion; the inflammation extending from the uterus into the connective tissue. The use of cold water vaginal injections, during or near the menstrual period; the various nefarious practices for the prevention of conception; the criminal interference with that function; and, lastly, a salpingitis may result in a pelvic abscess. I would not wish to be understood as having listed all the causes which serve to produce abscesses in the pelvis; these are merely a few examples that come readily to mind; I need not take time to mention others. If left to themselves, these abscesses generally rupture either into the peritoneum, the bladder, rectum, vagina, or even the uterus itself. I have seen two examples of the latter method of the spontaneous exit of pus from an abscess sac. In exceptional cases, the pus may burrow in the cellular tissue without making exit at all, ravaging the pelvis and finally ending the life of the patient by septicæmia and exhaustion. Sometimes several small collections of pus ultimately coalesce to form a larger single abscess. Rupture into the abdomen, of course, means death, and that speedily. If we could be assured that an abscess, in a given case, would certainly break through into the vagina, it would perhaps be justifiable practice to await the surgery of nature, provided the constitutional powers warranted such a delay. But observation and experience has sadly taught that the avenue through the vagina is as rarely sought spontaneously as any other; perhaps more so.

The expectant plan—that convenient name for idle waste of valuable time—has already been found inadequate here. Lives have been sacrificed through the glamour of its seductive influence. It is not many years since he would have been considered a bold surgeon, who would dare invade the pelvic cavity with a knife in search of pus; for, it must be admitted, there are few localities where it is more dangerous to cut than here. Competent authority asserts that there is not to be found in any other portion of the body, within the same extent of space, the same number of blood-vessels and nerves, as are distributed to the pelvic connective tissue. Nevertheless, pus is as much an enemy of the economy here as elsewhere; and I am persuaded that the soundest surgical principles would dictate the *early* artificial evacuation of a pelvic abscess. The difficulty, in many cases, lies in the way of diagnosis. It is not always possible to tell, with certainty, whether there is pent-up fluid lurking in some nook or cranny of the pelvic cavity; still less is it within the diagnostic ability of the most skilful touch to differentiate between serum and pus in all cases, even when once a deposit is found. But when the diagnosis of the presence of pus is made clear, we may not, I asseverate, hold off our hands a single day without jeopardy to the patient, and danger to our reputations as well; for should the abscess, meanwhile, rupture into the peritoneum, then surely would both patient and reputation be "in one red burial blent." This direful accident—minus the reputation—once happened to Sir James Y. Simpson. Thomas relates that Sir James saw a patient suffering with pelvic abscess one day with Dr. Zeigler. Feeling sure that it would soon discharge, they waited a day, when, to their surprise, it had burst into the peritoneum.

Having determined upon the necessity of an operation in a case of pelvic abscess, how shall it be performed? becomes a pertinent inquiry. If the case is one favorable for operation through the vagina, we have a number of different methods to choose from. There is the knife, the aspirator needle, and the thermo-cautery, the use of latter having been advocated by some surgeons, who would thereby avoid the danger of hæmorrhage. I should reject this instrument for the following reasons: 1st—If the pus sac should be deeply located, requiring much depth of incision to reach it, the dissection with the cautery would be coarse and unsurgical; besides, it would create a large slough, that would embarrass the after treatment. 2d—If the abscess walls were thin, and within easy reach, the danger of hæmorrhage would be too inconsiderable to establish its claims to superiority over simpler and neater methods. Since the aspirator has come into vogue, it furnishes an ample, and at the same time a neat and simple method of evacuation in these cases.

The needle is readily thrust through the vaginal wall into the abscess, and after the pus is evacuated, the sac, by reversing the pressure, can be injected with an antiseptic fluid of some sort. This should be withdrawn, and the process repeated until the water returns clear. The opening should then be enlarged, either with a bistoury or an expanding steel dilator—the latter is preferred as being safest—and a drainage tube inserted. If the knife is used, the needle affords a proper guide. It is a good plan to pack the vagina with antiseptic cotton or marine lint to bring, by pressure, the abscess walls into apposition.

The cavity must now be washed out daily—perhaps several times a day—with an antiseptic fluid, the mercuric chloride perhaps, or carbolic water; and an iodoform emulsion thrown into the sac after each washing. Pardon me if I stop to pay some attention to details here, for, upon the after treatment wholly depends the successful management of the case. If the mercuric chloride is employed, 1 to 5,000 will be strong enough. I am persuaded that this solution has been used, in many cases, stronger than necessary; hence, the reports of poisoning and other direful results with it. I have found a glass syringe holding 2 or 3 ounces to serve the best purpose for washing out the abscess; it enables one to observe his work best, and to avoid the introduction of air into the cavity, as the nozzle fits snugly into the tube. The surgeon must, even though it be irksome, do this work himself. He alone, by reason of having so often explored the parts, is familiar with the topography of the abscess and its surroundings. He is better able to judge of the thickness of the walls of the sac, and of the tension they will endure, without danger of rupture. Then, too, he will surely know when the cavity is absolutely clean, and when the work is otherwise well done. Even a very competent nurse must not be entrusted with this duty. There must be absolute cleanliness, and the surgeon must be satisfied with nothing short of it. Many a case, otherwise skilfully ministered unto, has been sacrificed by neglect, or only indifferent attention in this particular. I am well aware, gentlemen, that I am addressing an audience of skilled medical men, who are more or less familiar with these details, many of which may, perhaps, seem commonplace; but it certainly can do no harm for us to review them again, and I plead their special importance in connection with the subject of our discourse, in mitigation of the offence.

In chronic abscesses, which do not yield with reasonable promptitude to this plan, Prof. Byford proposes to scrape the interior of the sac with the curette, having for its purpose the removal of certain tag-like projections, which hang from the abscess walls. These excrescences are pathological proliferations of the granulations, which retard or prevent the healing process, and are easily broken down by the finger or dull curette. He says that if these masses are not removed, the cure is not so prompt, and pyæmic fever continues longer; but if we curette them off and thoroughly cleanse the cavity of them, the offensive odor of the discharge will at once disappear.

He furthermore asserts that cicatricial changes occasionally occur in the lining membrane of these cavities, converting abscesses into cysts; that he has, in some cases, been able to trace the formation of cystic tumors from abscesses, by the absorption of inflammatory deposits, disappearance of hardness, and the metamorphosis of the abscess walls into cicatricial membrane. The pus then gives place to serum, and the abscess assumes the appearance of an encysted tumor, possessing the qualities of a cyst, which absorbs and exudes fluid.

This is certainly a most interesting pathological phenomenon, and it is well to bear in mind its possibility.

It is interesting to note in this connection that the late Prof. Brickell of New Orleans, some years ago, drew attention to the fact that he had observed two distinct forms of pelvic inflammation—the one phlegmonous and tending to or resulting in abscess or suppuration; the other serous, and tending to or resulting in an effusion of serum, with occasionally flakes of coagulable lymph combined. He takes the ground that pelvic serous effusions are much more common than is generally supposed; and that they demand the same treatment as collections of pus require, viz.: prompt evacuation, either through the vagina or by abdominal incision. He reports a number of cases in support of his views, successfully treated by surgical interference, and pronounces tentative measures valueless after the effusion occurs. It seems probable, however, that some of his cases were such as Prof. Byford describes as having been converted from abscesses into cysts.

There is still another important way of evacuating a pelvic abscess, which deserves consideration before I close. It is by laparotomy, and known as the method of Mr. Lawson Tait. The operation consists essentially in opening the abdominal wall, stitching the abscess sac to the margins of the incision, opening the sac freely, cleansing it thoroughly, and finally treating it with the drainage tube.

The question presents itself, when shall we operate through the vagina; and when should Tait's operation be made?

There can be no doubt—the presumption is reasonable—that each operation has its own proper sphere; but to fully outline the field of either is a difficult labor, one which I shall only essay to hint at, very briefly, at this time.

The greatest difficulties in the way of operating through the abdomen, are presented in those cases in which the abscesses are small and situated low down in the pelvic cavity. It is extremely difficult, in such cases, to stitch the abscess sac to the peritoneal margins of the incision; per contra, in these very cases, the vaginal operation is made with comparative ease. We might, therefore, conclude that the position and size of the accumulation has much to do with the selection of the operation.

For myself, I should say that when the tumor lies along the utero-vaginal junction, and does not extend above the pelvic brim, I would select the vaginal operation; but in cases in which I had to press hard against the vaginal vault to reach the lower portion of the sac, and in which its upper border was easily mapped out above the brim, I would employ abdominal section.

Permit me to say, in conclusion, that I have purposely refrained from entering into Mr. Tait's interesting field of tubal diseases, with its various subdivisions of pyo- hydro- and hemato-salpynx, limiting myself to the consideration of pelvic abscess as it occurs in the cellular tissue. What I have said in regard to the evacuation of pelvic abscesses by laparotomy, refers simply to Tait's method of operating in the form of disease I have been considering, and not to the Hegar-Tait operation, which has for its special object the removal of the uterine appendages, when diseased.

When the modest Kentucky surgeon, in the year 1809, by his skill and courage, boldly opened Mrs. Crawford's abdomen, and successfully delivered her of a large ovarian cyst, he laid the foundation for all the abdominal surgery which the world has since seen grow into such splendid proportions and achieve such magnificent results. If it were possible to even approximatively estimate the years which have been added to the life of woman by this one operation, it is probable that the aggregate would seem incredible.

Around this one stalwart example of heroic and devoted skill has been constructed a series of surgical achievements which have made scores of men famous, and given gynecological art a lasting claim to recognition as a cognate branch of medical science. And when, in future ages, students of medical science and literature shall examine the records of the nineteenth century, though the list of benefactors to woman will be a long one, they will find McDowell's name first, and near it, in conspicuous characters, emblazoned on the scroll of fame, the name of Lawson Tait.

306 Franklin Street.

